# Physics‐Guided Multi‐Channel Restoration for Efficient and Accurate Atomic Force Microscopy Imaging

**DOI:** 10.1002/advs.78289

**Published:** 2026-10-18

**Authors:** Jaeuk Sung, Hongseok Kim, Seungjae Heo, Jin Tae Moon, Jangwoo Ko, Panithan Sriboriboon, Dong‐Gyu Lee, Sanghyun Jo, Hee Han, Jungho Ryu, Woo Lee, Jinseong Heo, Hyun‐Cheol Song, Yunseok Kim

**Affiliations:** ^1^ School of Advanced Materials Science and Engineering Sungkyunkwan University Suwon Republic of Korea; ^2^ Department of Applied Data Science Sungkyunkwan University (SKKU) Suwon Republic of Korea; ^3^ Department of Semiconductor and Display Engineering Sungkyunkwan University (SKKU) Suwon Republic of Korea; ^4^ Department of Materials Science and Engineering Korea University Seoul Republic of Korea; ^5^ Samsung Advanced Institute of Technology Suwon Republic of Korea; ^6^ National Nano Fab Center (NNFC) Daejeon Republic of Korea; ^7^ School of Materials Science and Engineering Yeungnam University Gyeongsan Gyeongbuk Republic of Korea; ^8^ Korea Research Institute of Standards and Science (KRISS) Daejeon Republic of Korea

**Keywords:** atomic force microscopy, deep learning, electrostatic force microscopy, multi‐channel image restoration, piezoresponse force microscopy

## Abstract

Scientific imaging instruments often acquire multiple physically complementary signals within a single measurement. In atomic force microscopy (AFM), high‐throughput imaging requires higher scan speeds, however, image quality is fundamentally limited by multiple distortions. These artifacts are further amplified for sharp nano/microstructures, making accurate image restoration inherently challenging. Most deep learning‐based approaches rely on a single primary channel and improve performance by increasing model complexity or training data. Here, we introduce a physics‐guided multi‐channel input approach for AFM that utilizes co‐acquired auxiliary signals to improve image restoration efficiency and accuracy. We construct a four co‐acquired channel input, each containing complementary physical information. By integrating these channels into a lightweight convolutional neural network, the proposed approach significantly improves restoration quality with minimal additional parameters. At a scan rate of 7.0 Hz, the four‐channel DnCNN achieved higher reconstruction accuracy than a single‐channel Restormer with approximately 47 times as many parameters, while providing approximately 24 times faster inference. Across the evaluated EFM and PFM conditions, four‐channel inputs reduced the training‐patch requirement by approximately two to three times at matched PSNR.

## Graphical abstract

Schematic figure of restoring noisy atomic force micrscopy (AFM) image via physics‐guided multi‐channel restoration. The lighter neural network models with multiple‐channel input from auxiliary signals outperforms heavier model with single‐channel input.

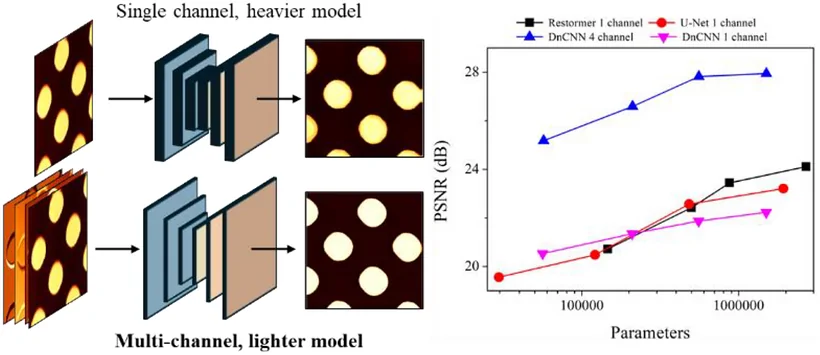

## 1 Introduction

Scientific imaging instruments routinely acquire multiple measurement channels during a single data acquisition. For example, magnetic resonance imaging (MRI) systems acquire multiple contrast weightings in a single session, multi‐spectral remote sensing satellites simultaneously record visible and infrared bands, and scanning electron microscopes (SEM) capture secondary and backscattered electron signals in parallel. Despite the availability of such complementary data sources, most deep learning approaches for image restoration rely on a single primary input channel and pursue quality improvement through architecture scaling or increased training data [1, 2, 3, 4, 5, 6]. Acquiring high‐quality training data requires extended instrument time, while deploying complex architectures increases computational cost. These constraints limit practical adoption in resource‐constrained laboratory settings.

Recently, the feasibility of enhancing image restoration by using auxiliary data sources has been successfully demonstrated across several imaging instruments, including remote sensing, MRI, fluorescence microscopy, and SEM [7, 8, 9, 10, 11, 12]. These auxiliary signals encode information about the degradation that is not contained in the primary channel and cannot be recovered through increasing model complexity alone. Physics‐guided approaches further show that incorporating domain knowledge at the input level can provide improvements from larger architectures [13]. Moreover, most auxiliary information can be acquired with minimal additional experimental time or cost. Despite this cross‐domain evidence, to the best of our knowledge, atomic force microscopy (AFM) has not explored co‐acquired signals for deep learning‐based image restoration, even though AFM measurements inherently record multiple physically complementary information [14, 15, 16].

In AFM, high‐throughput imaging requires increased scan speeds, However, image quality is limited by multiple sources of distortion, including feedback bandwidth limitations, tip‐sample convolution, drift, and directional asymmetry. These artifacts are further amplified in nano/microstructures with sharp topographic features, making accurate image restoration particularly challenging. When the scan speed exceeds the feedback bandwidth, the controller cannot adequately follow rapid topographic changes. At ascending step edges, the tip collides with the surface before the feedback loop responds, producing overshoot artifacts, whereas at descending step edges, the tip loses contact and parachutes above the surface, generating undershoot artifacts [17, 18, 19, 20, 21]. These effects become more pronounced at high scan speeds and ultimately limit measurement throughput across applications ranging from fundamental surface science to nanometer‐precision industrial metrology [22, 23, 24, 25]. Accordingly, scan speed‐induced artifacts provide a well‐defined and practically relevant testbed for evaluating image restoration strategies.

To address image restoration quality, deep learning approaches have progressed from relatively simple deep neural networks (DNNs) and convolutional neural networks (CNNs) to more advanced architectures such as generative adversarial networks (GANs) and transformers [26, 27, 28, 29, 30]. Although these architectures improve restoration quality, they often require substantially greater training time and computation recourses. Increasing the size of training datasets provides an alternative pathway, but this also demands additional training and experimental time. Consequently, improving performance solely through model complexity or data scaling presents practical limitations.

Here, we propose a physics‐guided multi‐channel input approach for AFM image restoration as an alternative to blind single channel denoising, with the goal of reducing both training time and computer cost while improving restoration quality. Instead of relying on increasingly complex models to extract information from a single input, the network utilizes multiple co‐acquired signals that contain complementary physical information related to the measurement process. Especially, we construct a four channel input consisting of trace topography, the trace–retrace topography difference, the trace error signal, and the retrace error signal. Because parachuting and collision artifacts occur at opposite edges depending on the scan direction and become more pronounced at high scan speeds, trace and retrace images contain complementary artifact signatures. The trace‐retrace difference captures this directional asymmetry at structural edges and serves as a spatially resolved artifact indicator of image degradation, while approaching zero in flat regions. In addition, the feedback loop generates an error signal at each pixel, representing the residual deflection that the controller fails to correct. This error signal contains high‐frequency surface features that are not present in the topography channel. Importantly, these auxiliary signals are directly linked to the underlying degradation mechanisms and can be obtained simultaneously during AFM imaging without additional measurement time or hardware modification.

Our approach concatenates four input channels while preserving model simplicity, since adding input channels modifies only the first layer of a deep neural network and introduces negligible parameter overhead. By integrating physically meaningful signals into a lightweight convolutional neural network, the proposed method improves restoration quality. In addition, it improves data efficiency by reducing the acquisition time required to reach comparable image quality. At a 7.0 Hz scan speed, a four‐channel DnCNN with 57K parameters reached approximately 25.21 dB, exceeding the 24.11 dB obtained by the 2686 K‐parameter single‐channel Restormer by 1.10 dB. The four‐channel model therefore used 47.1‐fold fewer parameters and required 1.13 ms per 64 × 64 patch, compared with 27.68 ms for the Restormer, corresponding to a 24.5‐fold inference‐speed advantage. Across EFM and PFM, the submitted patch‐fraction analysis showed 2.1–3.0‐fold higher training‐data efficiency with four‐channel input. Furthermore, the same approach was evaluated across topographic AFM, electrostatic force microscopy (EFM), and piezoresponse force microscopy (PFM), demonstrating its applicability beyond conventional topographic imaging. Overall, this work shows that incorporating physically meaningful auxiliary signals at the input level provides a more effective and practical alternative to increasing model complexity for scientific image restoration.

## 2 Results and Discussion

Figure 1 shows an overview of the physics‐guided multi‐channel input approach. Under certain scanning conditions, particularly at high scan speeds, the AFM feedback loop increasingly fails to track rapid topographic changes, resulting in collision artifacts at ascending step edges and parachuting artifacts at descending step edges. Micro‐patterned structures, which are prone to high scan speed artifacts, were chosen as the test specimen to examine the restoration of AFM topography images. For topographic AFM image restoration, we designed a multi‐channel input denoising convolutional neural network (DnCNN) architecture (Figure S1) [1]. Four signals (trace topography, trace‐retrace topography difference, trace error signal, and retrace error signal) are concatenated along the channel dimension and provided as input to the network for image restoration. The trace topography serves as the primary signal, while the other input channels provide auxiliary information. The trace‐retrace topography difference shows scan direction dependent parachuting and collision artifacts, whereas the trace and retrace error signals present feedback residuals that capture high‐frequency features missed by the feedback loop. The performance of multi‐channel input DnCNN architecture was compared with that of a single‐channel input Restormer architecture, which is more complex and requires heavier computations. (Details are available in the method section)

**FIGURE 1:**
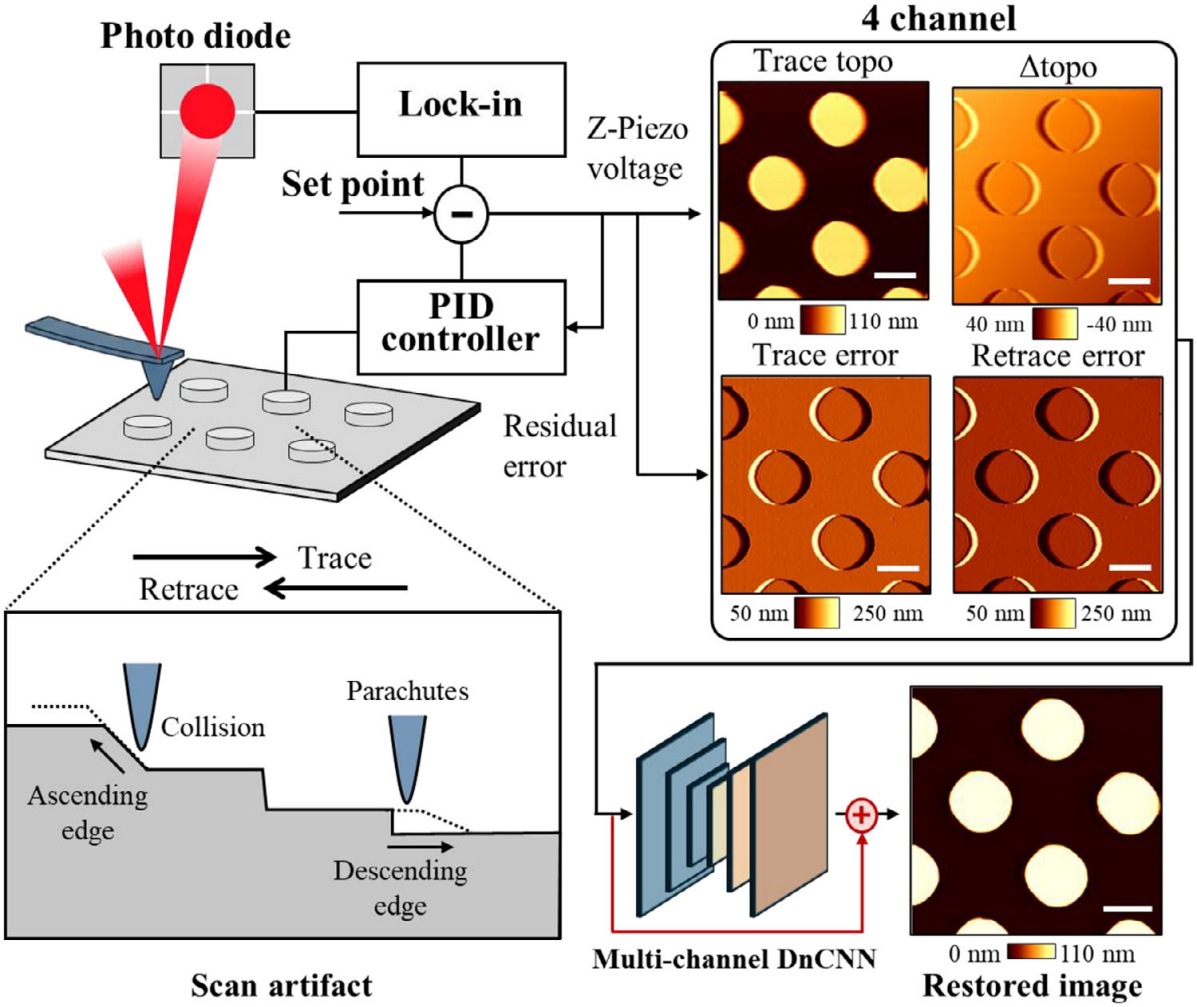
Schematics of AFM scanning artifacts and physics‐guided multi‐channel input approach for image restoration. At high scan speeds, the feedback loop can fail to track rapid topographic changes, producing collision artifacts at ascending edges and parachuting artifacts at descending edges. Noisy input image is restored with multi‐channel input constructed from four co‐acquired AFM channels consisting of trace topography (topo), trace‐retrace topography difference (Δtopo), trace error signal, and retrace error signal images. Images were acquired in non‐contact topographic AFM mode using an NX‐10 with a Multi75E‐G probe at 7.0 Hz and a feedback gain setting of 1.0. All scale bars are 2 µm.

The effect of scan speed on image quality and error signal was examined. Figure 2a presents trace topography and trace error images of micro‐patterned structures acquired at scan speeds from 1.0 to 7.0 Hz. As the scan speed increases, collision and parachuting artifacts at step edges become progressively more pronounced. Correspondingly, the error signal amplitude increases, indicating larger feedback residuals at higher scan speeds. This behavior indicates that the error signal carries progressively more restoration‐relevant information as artifacts become severe.

**FIGURE 2:**
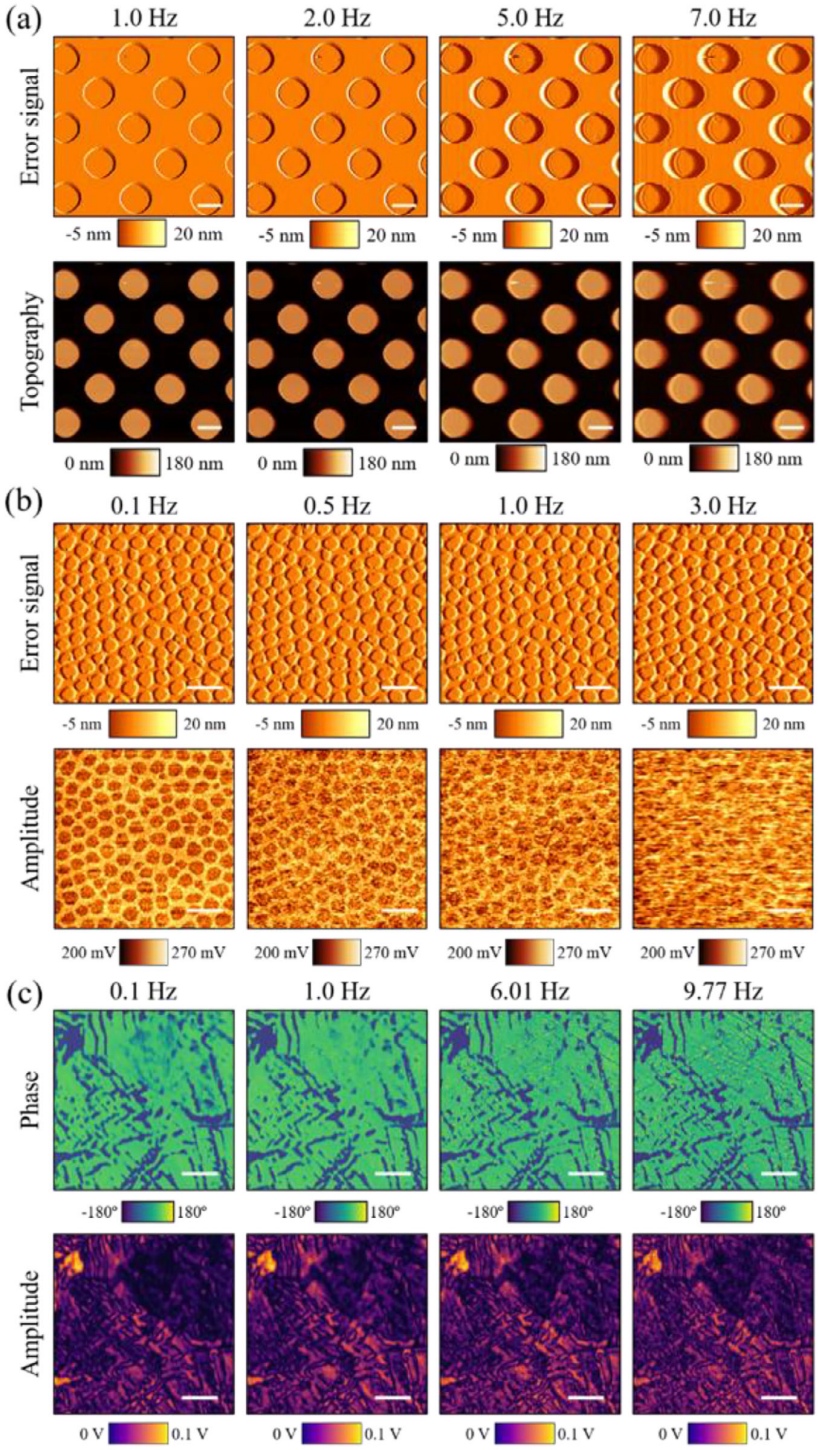
(a) AFM trace error signal and trace topography images of micro‐patterned structures acquired at varying scan speeds (1.0, 2.0, 5.0, and 7.0 Hz). Collision and parachuting artifacts increase progressively with increasing scan speed, accompanied by a corresponding increase in error signal amplitude, which provides increasingly valuable auxiliary information. (b) EFM trace error signal and trace amplitude images of nanocapacitors acquired at varying scan speeds (0.1, 0.5, 1.0, and 3.0 Hz). Random noise artifacts increase with increasing scan speed, while the error signal continue to capture nanocapacitor structures, providing valuable auxiliary information. (c) PFM trace phase and trace amplitude images of ceramic material acquired at varying scan speeds (0.10, 1.00, 6.01, and 9.77 Hz). Random noise and surface topography induced artifacts become more visible at increasing scan speed. Topographic AFM was measured with non‐contact mode NX‐10/Multi75E‐G. EFM was measured with dual‐pass lift‐mode EFM, lift height 50 nm. PFM was measured with MFP3D, AC excitation 2.5 V at 17 kHz. The instrument‐reported feedback gain setting was 1.0 for each measurement series. All scale bars are 2 µm.

The advantage of multi‐channel input was further cross‐validated using EFM and PFM modes (Figure 2; Figure S2 and Table S1). In these modalities, image degradation arises from non‐topographic sources, including electronic noise, lock‐in artifacts, and time constant effects, producing spatially distributed distortions across the image. Figure 2b shows trace amplitude and trace error images of nanocapacitors measured by EFM at scan speeds from 0.1 to 3.0 Hz. At high scan speeds, the amplitude images are significantly degraded by random noise, whereas the error signal retains clear structural features of the nanocapacitor, which can provide complementary spatial information for image restoration. The scan speed‐dependent PFM measurements are presented in Figure 2c. The phase‐trace and amplitude‐trace images were acquired over the same field of view at 0.10, 1.00, 6.01, and 9.77 Hz. At the lowest scan speed (0.1 Hz), the ferroelectric domain morphology is clearly resolved in both channels. With increasing scan speed, the local domain contrast becomes less distinct from random noise artifacts, and topography‐induced artifacts become more and more visible. These observations confirm that auxiliary channels encode information about artifact location and magnitude that is not accessible from the primary channel alone, particularly under high‐speed imaging conditions where restoration is most critical. For EFM and PFM images, DnCNN showed limited performance for restoration capabilities. Unlike topographic artifacts localized at step edges, these degradations require multi‐scale feature extraction for effective restoration. Thus, a nonlinear activation‐free network (NAFNet)‐based U‐Net architecture was adopted for the cross‐modal experiments (Figure S2) [31]. For PFM, the phase and amplitude signals are intrinsically coupled through the piezoelectric response, meaning that amplitude images contain information about domain boundaries that aids phase restoration [32, 33, 34, 35]. Detailed experiment conditions are available in method sections and supporting information.

Multi‐channel input improves reconstruction quality for both topography and EFM images. Figure 3a shows topographic image restoration of micro‐patterned structures acquired at 7.0 Hz. The single‐channel model represents conventional image restoration with a single primary channel as input. The two‐channel model is a pre‐defined set of primary‐auxiliary signal input used to evaluate the contribution of a physically relevant auxiliary signals. Alternative channel combinations, when evaluated, are therefore reported separately rather than pooled into a generic two‐channel result. The single‐channel model recovers overall surface morphology but produces blurred edges and residual scan speed artifacts. In contrast, the four‐channel model recovers sharp step edges that closely match the slow‐scan ground truth. Figure 3b shows EFM amplitude restoration of nanocapacitor structures acquired at 3.0 Hz using the NAFNet architecture. The single‐channel model fails to resolve individual nanocapacitor features, while the four‐channel model clearly recovers the structural pattern. For topography, PSNR increases from 21.87 dB (single channel) to 27.58 dB (four channel) at 1501 K parameters (Figure 3c). For EFM amplitude, the four‐channel model similarly outperforms single‐ and two‐channel models across all tested model sizes (Figure 3c). The four‐channel models also converge faster during training, reaching their final SSIM plateau within 20 epochs compared to 40 epochs for single‐channel models (Figure S5). The multi‐channel PSNR gain increases with artifact severity, scaling from +0.14 dB at 1.0 Hz to +5.71 dB at 7.0 Hz (Figure S7), confirming that auxiliary channels become increasingly informative under conditions where restoration is most needed. To resolve the contribution of individual auxiliary signals, all eight combinations of the trace–retrace topography difference (D), trace error (E_t_), and retrace error (E_r_) were evaluated while retaining trace topography (T). The mean PSNR increased from 21.28 dB for T alone to 25.57 dB for the complete four‐channel input. Removing D, E_t_, or E_r_ reduced PSNR to 24.80, 24.75, and 24.53 dB, respectively, with the largest reduction observed upon removal of E_r_. (Figure S8) Four‐channel controls using repeated primary images, mismatched auxiliary signals, and Gaussian noise matched to the auxiliary channel means and standard deviations achieved 21.36, 19.90, and 20.12 dB, respectively. (Figure S9) These controls support the contribution of spatially corresponding measurement information beyond increased input dimensionality.

**FIGURE 3:**
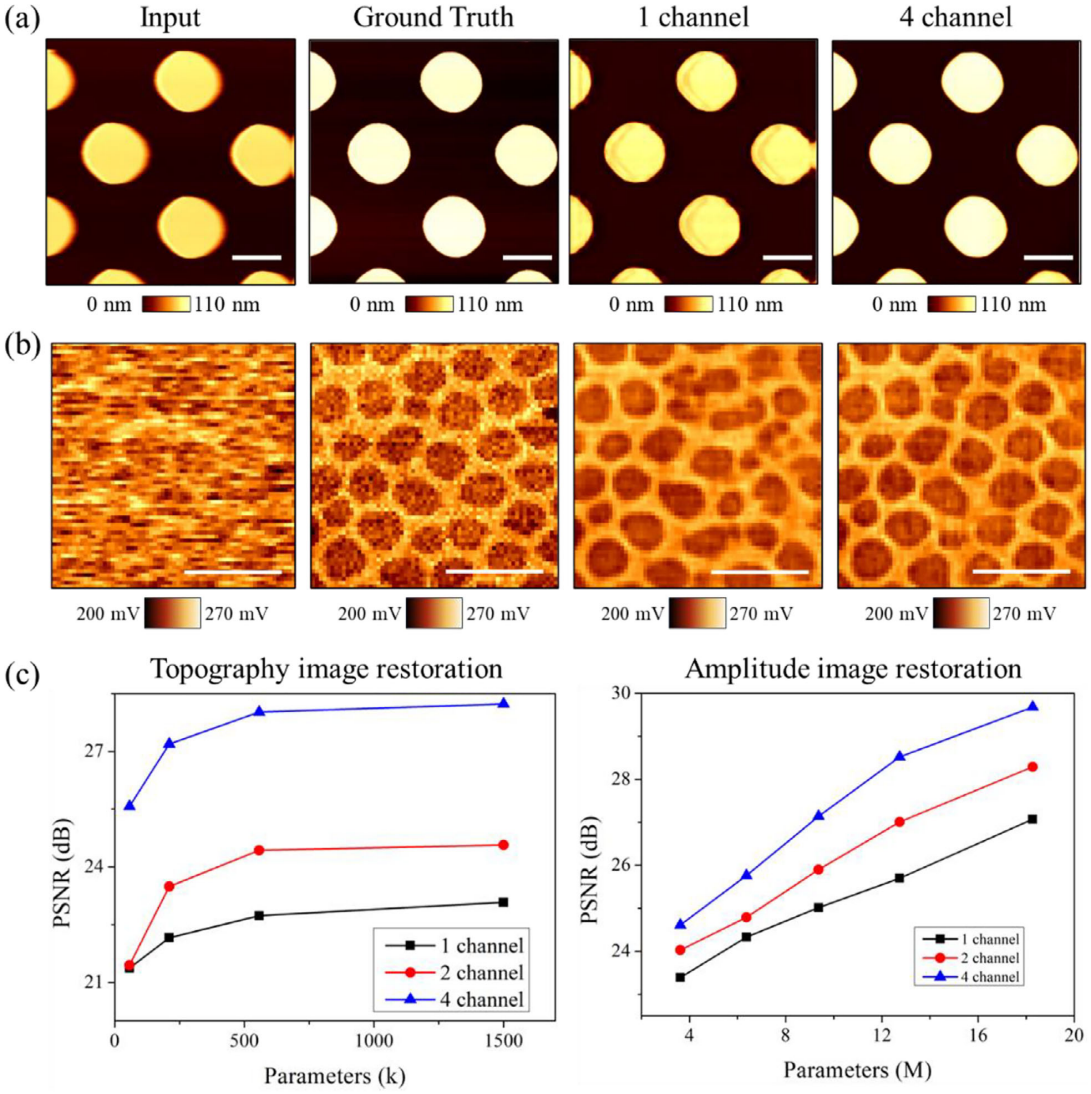
Multi‐channel AFM restoration results and channel number‐dependent analysis. (a) Visual comparison of input, ground truth, single‐channel, and four‐channel inputs for micro‐patterned structure AFM topography images acquired at 7.0 Hz using DnCNN. The four‐channel model recovers fine surface features that are not resolved by the single‐channel model. (b) Visual comparison of input, ground truth, single‐channel, and four‐channel inputs for nanocapacitor EFM amplitude images acquired at 3.0 Hz using NAFNet architecture. Similar to topography images, the four‐channel model recovers features that are not resolved by the single‐channel model. (c) Image restoration quality as a function of channel number for AFM topography and EFM amplitude images acquired at 7.0 Hz and 3.0 Hz, respectively, with different model sizes. For topography, 1 channel = trace topography and 2 channel = trace topography and trace–retrace topography difference, and 4 channel = trace topography, trace–retrace topography difference, trace and retrace feedback‐error signals. For EFM amplitude, 1 channel = trace amplitude, 2 channel = trace amplitude and retrace amplitude, and 4 channel = trace and retrace amplitude and error signal. Topographic AFM was measured with non‐contact mode NX‐10/Multi75E‐G. EFM was measured with dual‐pass lift‐mode EFM, lift height 50 nm. All scale bars are 2 µm.

Beyond PSNR and SSIM, structural fidelity was evaluated using boundaries extracted with thresholds determined from the corresponding ground truth images. With a two‐pixel matching tolerance, the boundary F1 score increased from 0.444 to 1.000 for topographic step boundaries, from 0.917 to 0.961 for PFM domain walls, and from 0.968 to 0.982 for EFM feature boundaries when moving from single channel to four channel restoration. The corresponding false‐boundary fractions decreased from 0.527 to 0.000, from 0.102 to 0.060, and from 0.053 to 0.033, respectively. These results illustrate improved boundary agreement with the reference measurements. Detailed methods, boundary overlays, and quantitative results are provided in the Supporting Information.

We further examined PFM phase restoration on a polycrystalline lead zirconate titanate (PZT) thin film using models fine‐tuned separately for four lock‐in time constants (Figures S14 and S15). The four‐channel reconstruction improved PSNR by 0.35–0.80 dB over single channel reconstruction, with corresponding improvements in SSIM and Pearson correlation. This shows that the multi‐channel PFM phase image restoration can expand beyond KBT‐BT‐NBT materials, and be generalized to different materials.

The multi‐channel input to a lightweight model outperforms single‐channel architecture scaling at every parameter count tested. We compared the four‐channel DnCNN against single‐channel U‐Net and single‐channel Restormer, each representing increasing levels of architectural complexity [4, 29]. U‐Net was selected for its skip‐connection‐based denoising capability, and Restormer was selected as the current state‐of‐the‐art architecture for AFM image restoration. As shown in Figure 4a, a four‐channel DnCNN with 210 K parameters produces higher‐quality restoration than both a single‐channel U‐Net with 483 K parameters and a single‐channel Restormer with 499K parameters, despite having two to three times fewer parameters. Line profiles along a cross‐section (Figure 4c) confirm the four channel DnCNN best matches the ground truth surface topography, with sharp transitions at step edges where both single channel models smooth over fine features. The model size dependent image restoration quality plot (Figure 4b) shows the four channel DnCNN dominates all single channel architectures at every parameter count: the smallest four‐channel DnCNN with 57K parameters (25.57 dB) outperforms the largest single‐channel Restormer with 2686 K parameters (24.11 dB), a 47‐fold parameter reduction with +1.46 dB quality improvement. This result demonstrates that exploiting co‐acquired auxiliary data enhances restoration quality more efficiently than scaling architectural complexity. This comparison involves changes in both architecture and input information and should not be interpreted as an architecture‐only comparison.

**FIGURE 4:**
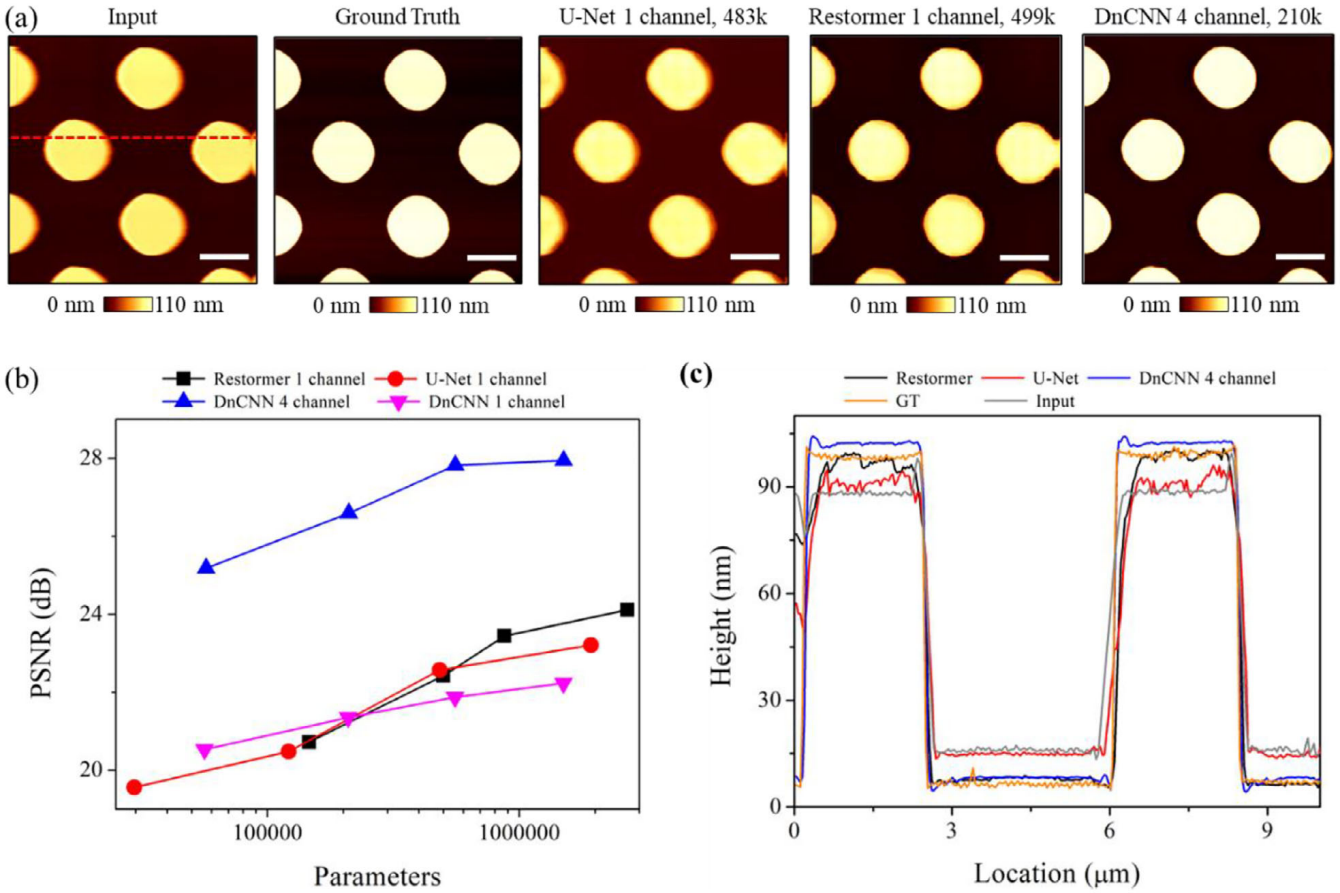
Physics‐guided multi‐channel input compared with architecture scaling for restoring AFM topography image acquired at 7.0 Hz. (a) The four channel DnCNN (210 K parameters) achieves higher‐quality restoration than single channel U‐Net (483 K) and single channel Restormer (499 K), despite having 2–3 times fewer parameters. (b) Peak Signal‐to‐Noise Ratio (PSNR) comparison across architectures confirms that the four channel DnCNN achieves the highest reconstruction quality at every model size, with four channel model with 57K parameters outperforming the largest single channel Restormer with 2686 K parameters. (c) Line profiles along the marked cross‐section show that four channel DnCNN best matches the ground truth (GT) surface topography. Topographic AFM images were acquired using an NX‐10 microscope (Park Systems) in non‐contact mode with a Multi75E‐G probe (Budget Sensors; nominal spring constant ≈ 3 N m^−^^1^). The input and reference images were acquired at scan rates of 7.0 and 0.5 Hz, respectively, with 256 × 256 pixels and an instrument‐reported feedback gain setting of 1.0. Images were acquired in non‐contact topographic AFM mode using an NX‐10 with a Multi75E‐G probe, at input/reference scan rates of 7.0/0.5 Hz and a feedback gain setting of 1.0. All scale bars are 2 µm.

**FIGURE 5:**
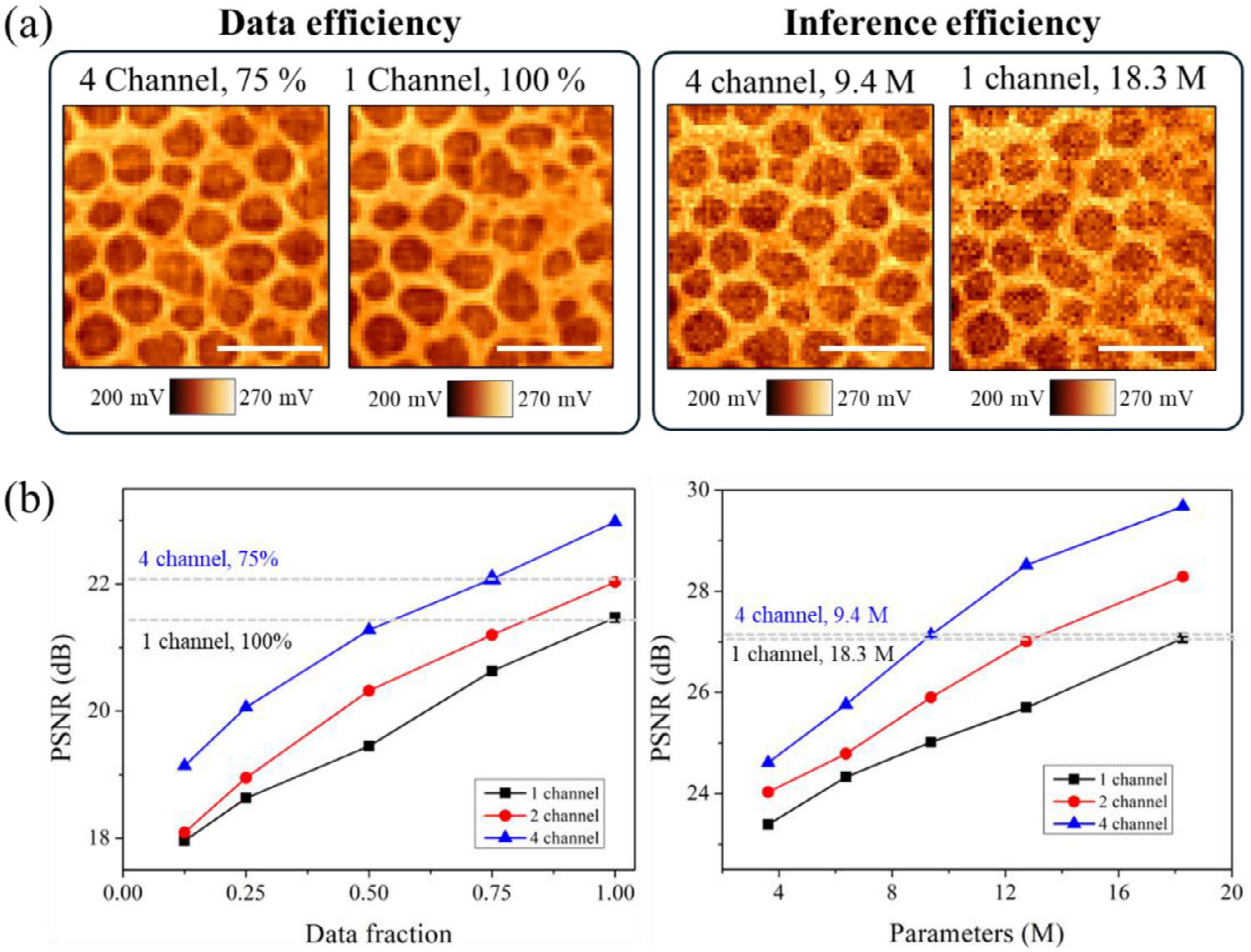
Data efficiency and inference efficiency examined with EFM amplitude image restoration for image acquired at 3.0 Hz. (a) Representative image restoration result for data efficiency and inference efficiency results. Data efficiency analysis shows that four‐channel model with 75% training data outperforms the single‐channel model with 100% of the data at equal model capacity (6.37M parameters). Inference efficiency analysis shows four channel model with 9.4M parameters outperform the single‐channel model with 18.3M parameters. (b) Peak signal‐to‐noise ratio (PSNR) as a function of training data fraction, showing that four‐channel model achieves performance equivalent to the single‐channel model with approximately half data. PSNR as a function of model size, confirming that multi‐channel input enables ∼2 times parameter reduction without quality loss. Images were acquired in dual‐pass lift‐mode EFM using an FX40 with a PPP‐EFM probe, at input/reference scan rates of 3.0/0.1 Hz, a lift height of 50 nm, and a feedback gain setting of 1.0. All scale bars are 1 µm.

We additionally examined single‐ and four‐channel configurations within the tested architecture families (Figure S18). The PSNR increased from 22.10 to 27.48 dB for DnCNN, from 23.01 to 25.65 dB for U‐Net, and from 22.91 to 26.86 dB for the parameter‐matched Restormer. These results support the benefit of co‐acquired auxiliary signals across the tested architectures. Conventional image restoraction were also additionally examined. The trace–retrace averaging achieved 18.77 dB, while Gaussian, median, Wiener, and non‐local‐means filtering achieved 17.48–17.56 dB, compared with 25.57 dB for the physical four‐channel DnCNN.

The data and inference efficiency of multi‐channel input was further examined using EFM amplitude images (Figure 5). The training‐data and parameter efficiency of multi‐channel EFM amplitude restoration was quantitatively evaluated at 3.0 Hz as shown in Figure 5b. At a fixed model capacity of 3.62M parameters, the four channel model trained with 25% of the available patches achieved a full‐image PSNR/SSIM of 20.34 dB/0.6743, comparable to or higher than the 20.13 dB/0.6486 obtained by the single channel model trained with 50% of the data. Linear interpolation indicated that the four‐channel model required approximately 2.1‐fold fewer training patches to achieve the same PSNR. In the parameter‐efficiency analysis, the 6.37M‐parameter four‐channel model achieved 25.76 dB/0.9125, comparable to the 25.70 dB/0.9119 of the 12.74M‐parameter single‐channel model. For three paired EFM fields, the four‐channel model improved PSNR by 1.21 dB and SSIM by 0.050 relative to the single‐channel model. These improvements demonstrate that auxiliary channels reduce the complexity of the restoration mapping, allowing smaller models and fewer training examples to achieve equivalent or superior performance. Cross‐modal validation on PFM extends these observations beyond scan speed artifacts. PFM phase images were degraded by varying scan speed and time constant, producing spatially distributed noise patterns distinct from topographic edge artifacts. Even for these different degradation mechanisms, the four‐channel model consistently outperforms the single‐channel model across all tested conditions (Figures S16 and S17).

Figure 6 quantifies the advantages of multi‐channel input in practical cost metrics. The PSNR as a function of inference time for DnCNN four‐channel, DnCNN single‐channel, and Restormer single‐channel at four model sizes is shown in Figure 6a. The four‐channel DnCNN dominates all single‐channel architectures across the full inference time range. The 57 k four‐channel DnCNN requires only 1.13 ms inference time, and outperforms a 2686 k single‐channel Restormer that requires 27.68 ms inference time by +1.10 dB, achieving 47‐fold parameter reduction and 24‐fold inference speedup simultaneously. Figure 6b shows ground‐truth experimental data acquisition time as a function of restored image quality for EFM and PFM. At matched image quality, the four‐channel model reaches the same PSNR with 35% less acquisition time for EFM scan speed noise, 25% less for PFM scan speed noise, and 38% less for PFM time constant noise. These savings translate directly to reduced instrument occupation time for laboratories acquiring training datasets. These results quantitatively show that experimental and computational time and cost are reduced by applying multi‐channel image restoration.

**FIGURE 6:**
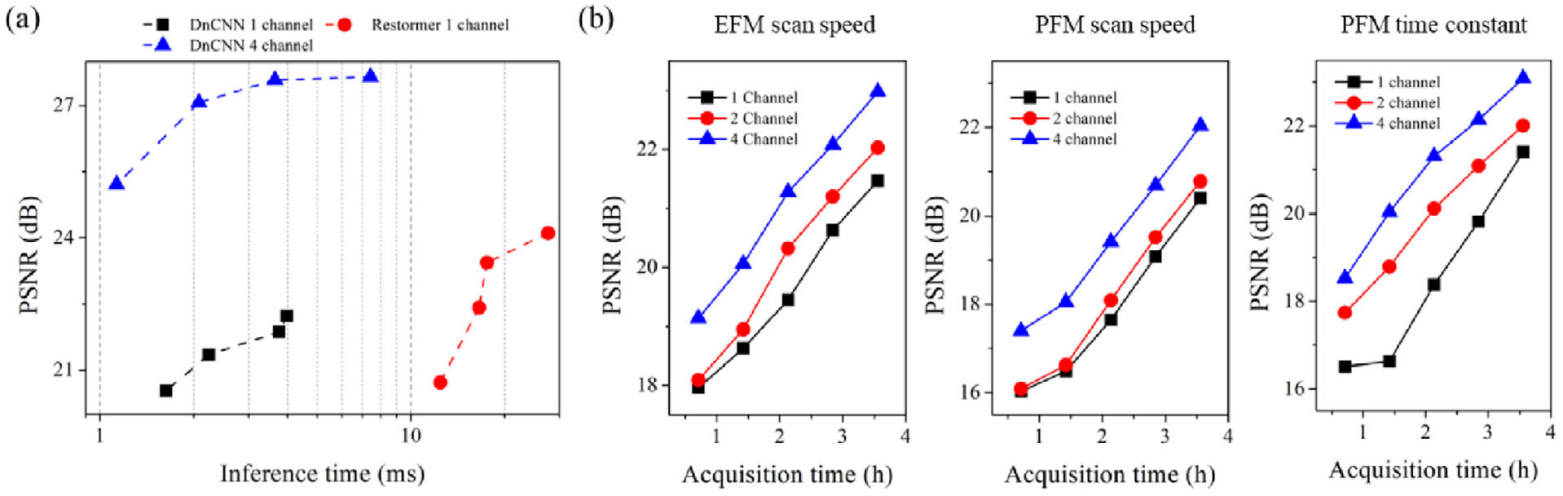
Practical benefit analysis of multi‐channel input design. (a) PSNR as a function of inference time for four‐channel DnCNN, single‐channel DnCNN, and single‐channel Restormer across four model sizes. The smallest four‐channel model (57K parameters, 1.13 ms) outperforms the largest single‐channel Restormer (2686 K parameters, 27.68 ms) by +1.10 dB with 47‐fold fewer parameters and 24‐fold faster inference. (b) PSNR as a function of ground truth acquisition time (hours) for EFM 3.0 Hz, PFM scan speed, and PFM time constant. Multi‐channel input reduces the required acquisition time by 35% for EFM, 25% for PFM scan speed, and 38% for PFM time constant.

The present framework depends on the quality, physical relevance, and spatial registration of the auxiliary channels. When an auxiliary signal is saturated, excessively noisy, misregistered, or degraded earlier or more severely than the primary signal, multi‐channel fusion may provide misleading information and can perform worse than the unprocessed input. The method may also fail for out‐of‐distribution samples, instruments, probes, or degradation mechanisms not represented in the training data, including severe drift, abrupt tip changes, and signal clipping. Accordingly, channel‐quality assessment and independent validation are required before quantitative use under new measurement conditions, followed by retraining or fine‐tuning when necessary.

## 3 Conclusions

This work demonstrates that input‐level fusion of co‐acquired AFM signals provides an efficient and practical route for image restoration. During AFM imaging, topographic edges introduce parachuting and collision artifacts with direction‐dependent signatures in trace and retrace data. These artifacts become more pronounced during high‐speed imaging conditions due to feedback limitations. Accordingly, the benefit of multi‐channel input increases with artifact severity. In contrast, conventional single channel approaches depend solely on topography, requiring the network to infer the degradation from incomplete observations, typically necessitating larger models and more training data.

Input‐level fusion directly incorporates physically meaningful auxiliary signals that capture directional asymmetry and feedback residuals, reducing ambiguity during training and improving data efficiency. The improvement in reconstruction quality becomes more evident as acquisition conditions become more challenging, highlighting a clear distinction from architecture scaling, which typically provides gradual improvements that are less sensitive to acquisition conditions. As a result, a lightweight multi‐channel model can surpass larger single‐channel architectures while introducing only minimal parameter overhead, achieving both improved accuracy and faster inference.

Cross‐modal results in EFM and PFM confirm that this mechanism extends beyond topography‐related artifacts. Despite distributed noise sources, paired trace and retrace signals still capture correlated degradation patterns, enabling comparable performance with reduced training data and model complexity.

Overall, auxiliary channels that are mechanistically linked to degradation provide information unavailable to single‐channel pipelines. Prioritizing physics‐guided channel selection and simple concatenation is more effective than increasing model size, as input information determines the upper bound on recoverable detail. Future work should extend this framework to additional AFM modalities and establish criteria for selecting auxiliary channels under varying experimental conditions. These efforts could enable robust application of multi‐channel restoration approaches under a wide range of experimental constraints.

## 4 Methods

### 4.1 Sample Preparation

For topography measurements, commercial silicon masters (Budget Sensors, Bulgaria, HS‐100MG) were used. Each master contains 2 × 2 µm protruding circular features spaced 2 µm apart. For PFM measurement, a textured KBT‐BT‐NBT tetragonal‐phase lead‐free ferroelectric ceramic material with the composition of (0.2($\frac{2}{3}$ K_0.5_Bi_0.5_TiO_3_–$\frac{1}{3}$ BaTiO_3_)‐0.8Na_0.5_Bi_0.5_TiO_3_) was fabricated following the method reported by Thakre et al. [36]. Additional PFM measurements were performed on a commercially available PZT thin film purchased from Korea Coating Materials & Components Co., Ltd. (KCMC, Korea), with a PZT/TiO_2_/Pt(111)/Ti/SiO_2_/Si(100) structure and corresponding layer thicknesses of 100 nm/10 nm/150 nm/10 nm/300 nm/525 µm, respectively. For EFM measurement, a Hf_0.5_Zr_0.5_O_2_ nanocapacitor was fabricated similar to previously reported methods [37]. A 10 nm‐thick Hf_0.5_Zr_0.5_O_2_ thin film was prepared using atomic layer deposition (ALD) with an anodized aluminum oxide (AAO) template with approximately 380 nm pore size as a shadow mask. A 30‐nm‐thick molybdenum top electrode was deposited via e‐beam evaporation through the AAO mask. A Mo/HZO/Si nanocapacitor was fabricated by mechanically removing the AAO mask. Finally, the nanocapacitor was annealed using a rapid thermal annealing process at the temperature of 350°C for 1 min.

### 4.2 AFM‐Topographic Measurements

AFM‐topographic measurements were carried out on a commercial atomic force microscope (NX‐10, Park Systems) equipped with Cr/Pt‐coated conductive tips (Multi75E‐G, Budget Sensors, nominal spring constant k ≈ 3 N/m). Although this probe has a conductive Cr/Pt coating, its electrical conductivity was not used and was not required for the present topography measurements. The measurement area was set to 10.0 × 10.0 – 30.0 × 30.0 µm^2^ with 256 × 256 pixels, and scan speeds varied from 0.5 Hz (ground truth) to 7.0 Hz (high scan speed input). All AFM measurements were performed in non‐contact mode.

### 4.3 PFM Measurements

PFM measurements were conducted using a commercial atomic force microscope (MFP3D, Asylum Research) equipped with Pt/Ir‐coated conductive tips (PPP‐NCHPt, Nanosensors, nominal spring constant k ≈ 42 N/m) and a lock‐in amplifier (MFLI, Zurich Instruments). For KBT‐BT‐NBT, the measurement area was set to 10.0 × 10.0 µm^2^ and 256 × 256 pixels. An AC bias voltage of 2.5 V with 17 kHz was applied to the tip for piezoresponse excitation. Lock‐in amplifier parameters were systematically varied to generate input and ground truth data. Time constants varied from 3.823 ms (ground truth) to 0.1 ms (time constant input), while scan speeds varied from 0.1 Hz (ground truth) to 9.77 Hz (high scan speed input). For PZT, measurements were performed under the same conditions described above, except for the scan area and AC bias voltage. The scan area was set to 5.0 × 5.0 µm^2^, and an AC bias voltage of 1.5 V was applied to the tip.

### 4.4 EFM Measurements

EFM measurements were carried out on a commercial atomic force microscope (FX40, Park Systems) equipped with Pt/Ir‐coated conductive tips (PPP‐EFM). The measurement area was set to 10.0 × 10.0 µm^2^ with 256 × 256 pixels, and scan speeds varied from 0.1 Hz (ground truth) to 3.0 Hz (high scan speed input). A DC bias of −2 V with an AC voltage of 2 V at 17 kHz was applied to the tip to induce an electrostatic force, which was detected by the cantilever response. All EFM measurements were performed in lift‐mode using a dual‐pass scanning method with a lift height of 50 nm.

### 4.5 Dataset Construction and Preprocessing for Topographic AFM, PFM, and EFM

For topographic AFM image restoration, we collected 11 trace–retrace image pairs at 256 × 256 pixels. Each pair consists of a forward (trace) and backward (retrace) scan at high speed (7.0 Hz) with corresponding slow‐scan ground truth (0.5 Hz). Each sample provides four signals: trace topography, retrace topography, trace error, and retrace error. We extracted 64 × 64 patches with stride 32, yielding 539 base patches per configuration. Standard augmentation including horizontal flip, vertical flip, and four 90‐degree rotations expanded the effective training set to 6896 patches (Table S2). For EFM, we collected 5 image pairs at 256 × 256 pixels, with input at 3.0 Hz scan speed and ground truth at 0.1 Hz. Available signals include amplitude trace/retrace and error signal trace/retrace. For PFM, we collected 5 image pairs with two degradation conditions: scan speed (9.77 Hz input, 0.1 Hz ground truth) and time constant (0.1 ms input, 3.823 ms ground truth). Available PFM signals include amplitude trace/retrace and phase trace/retrace. Both EFM and PFM datasets were processed identically: 64 × 64 patches with stride 32, augmented to 3136 training patches per condition (Tables S1 and S2). Before patch extraction, the input images were converted to floating‐point arrays and processed using modality‐specific intensity scaling. For topographic AFM, planar backgrounds were removed independently from the trace and retrace topography images using a plane fitted to the mean values of four corner regions, each comprising 5 × 5 pixels. Each flattened topography image and each error‐signal image was then independently min–max normalized to [0, 1]. The topography‐difference channel was calculated by subtracting the normalized retrace topography from the normalized trace topography, without subsequent rescaling. This channel therefore represents a dimensionless trace–retrace discrepancy rather than an absolute height difference. For PFM, each amplitude and phase channel was independently min–max normalized to [0, 1].

### 4.6 Multi‐Channel Input Configuration

Multi‐channel input was used for image restoration with different AFM modality. The term image restoration denotes the correction of dynamic acquisition artifacts caused primarily by finite feedback or detection bandwidth. It does not refer to general drift correction, tip‐shape deconvolution, or full inverse imaging. For AFM topographic image restoration, the deep learning model receives four channels concatenated along the channel dimension: (1) trace topography, (2) trace‐retrace topography difference, (3) trace error signal, and (4) retrace error signal. We also evaluated intermediate configurations: single channel (trace topography only) as the baseline, and two channel (trace topography concatenated with trace–retrace difference) to quantify the incremental contribution of each information source. For EFM amplitude image restoration, deep learning model receives four channels concatenated along the channel dimension: (1) trace amplitude, (2) retrace amplitude, (3) trace error signal, and (4) retrace error signal. For PFM phase image restoration, deep learning model receives four channels concatenated along the channel dimension: (1) trace phase, (2) retrace phase, (3) trace amplitude, and (4) retrace amplitude. For multi‐channel inputs, the channels were concatenated and fused through the first 3 × 3 convolutional layer of DnCNN. The network then predicts a single‐channel residual that is added to the trace topography. No manual channel weighting or separate fusion module was used.

### 4.7 Dncnn Architecture

For topographic image restoration, we adopted a DnCNN with residual learning (Figure S1). The network consists of convolutional layers with batch normalization and ReLU activation. The first layer accepts the multi‐channel input (single, two, or four channels) through a standard convolutional kernel, and the final layer outputs a single‐channel residual through a sigmoid activation, which is added to the trace topography via a skip connection to produce the restored image. We evaluated four model sizes: tiny (8 layers, 32 features, 57 K parameters), small (12 layers, 48 features, 210 K parameters), mid (17 layers, 64 features, 558 K parameters), and large (20 layers, 96 features, 1501 K parameters). The parameter difference between single‐channel and four‐channel DnCNN is confined to the first convolutional layer and is at most 1.5% of total parameters for the smallest model and less than 0.5% for all others.

### 4.8 NAFNet Architecture

For cross‐modal experiments on EFM and PFM, we adopted a nonlinear activation‐free network (NAFNet)‐based U‐Net architecture (Figure S2). Each building block consists of two sub‐blocks with residual connections and learnable scaling parameters. The first sub‐block performs spatial mixing through a sequence of LayerNorm, 1 × 1 convolution, 3 × 3 depth‐wise convolution, SimpleGate (channel‐split element‐wise product that replaces conventional nonlinear activations), simplified channel attention (SCA, global average pooling followed by a 1 × 1 convolution), and a 1 × 1 output projection. The second sub‐block performs channel mixing through LayerNorm, 1 × 1 convolution, 3 × 3 depth‐wise convolution, SimpleGate, and a 1 × 1 output projection. The overall architecture follows a four‐level encoder–decoder structure with three downsampling and upsampling stages. Downsampling is performed by a 3 × 3 convolution followed by PixelUnshuffle with a factor of 2, and upsampling by a 3 × 3 convolution followed by PixelShuffle with a factor of 2. Skip connections concatenate encoder and decoder feature maps at each level, followed by a 1 × 1 convolution for channel dimension reduction. A 3 × 3 convolution serves as the patch embedding layer at the input, and the output is produced through a 3 × 3 convolution combined with the input trace channel via residual learning. Feature dimensions double at each encoder level (dim → 2×dim → 4×dim → 8×dim). The architecture accepts multi‐channel inputs (1, 2, or four channels) and outputs the restored signal through a skip connection from the first input channel. We evaluated five model sizes: AFM‐T (dim 24, [2,4,4,6] blocks, 2 refinement blocks, 3.62M parameters), AFM‐XS (dim 32, [2,4,4,6] blocks, 2 refinement blocks, 6.37M parameters), AFM‐S (dim 32, [4,6,6,8] blocks, 4 refinement blocks, 7.40M parameters), AFM‐M (dim 40, [4,6,6,8] blocks, 4 refinement blocks, 12.74M parameters), and Base (dim 48, [4,6,6,8] blocks, 4 refinement blocks, 18.27M parameters).

### 4.9 U‐Net Architecture

For the U‐Net architecture, we implemented a standard encoder–decoder architecture with skip connections. Each encoder level consists of a double convolution block (two successive 3 × 3 Conv2d–BatchNorm–ReLU sequences), followed by 2 × 2 max‐pooling for spatial downsampling. The symmetric decoder upsamples feature maps using 2 × 2 transposed convolutions and concatenates them with the corresponding encoder feature maps through skip connections before applying the same double convolution block. The final layer projects the feature maps to a single channel through a 1 × 1 convolution with sigmoid activation, and the output is combined with the trace topography input via residual learning, identical to the DnCNN formulation. Channel dimensions double at each encoder level (base → 2×base → 4×base → 8×base). We evaluated four single‐channel model sizes: tiny (base channels 8, depth 3, 30 K parameters), small (base channels 8, depth 4, 122 K parameters), mid (base channels 16, depth 4, 483 K parameters), and large (base channels 32, depth 4, 1929 K parameters).

### 4.10 Restormer Architecture

For the Restormer baseline, we implemented a Transformer‐based encoder–decoder architecture following the original design. Each Transformer block consists of multi‐Dconv head transposed attention (MDTA) and a gated‐Dconv feed‐forward network (GDFN) with layer normalization and residual connections. In MDTA, queries, keys, and values are generated through a 1 × 1 convolution followed by a 3 × 3 depth‐wise convolution, and attention is computed across the channel dimension with a learnable temperature parameter per head, reducing computational complexity from quadratic to linear with respect to spatial resolution. The GDFN employs a gated mechanism in which the projected features (expansion factor 2.66) are split into two branches after a 3 × 3 depth‐wise convolution, and one branch is gated by the GELU activation of the other. The architecture follows a three‐level encoder–decoder structure with stride‐2 convolutions for downsampling and transposed convolutions for upsampling; skip connections are realized by channel‐wise concatenation followed by 1 × 1 convolution for dimension reduction. A 3 × 3 convolution serves as the patch embedding layer at the input, and the output is produced through a 3 × 3 convolution with sigmoid activation combined with the trace topography via residual learning. We evaluated four single channel model sizes: tiny (dim 16, [1,1,1,1] blocks, [1,1,2,4] heads, 146K parameters), small (dim 24, [1,2,2,2] blocks, [1,2,4,4] heads, 499K parameters), mid (dim 32, [1,2,2,2] blocks, [1,2,4,4] heads, 874 K parameters), and large (dim 48, [2,3,3,4] blocks, [1,2,4,8] heads, 2686 K parameters).

### 4.11 Training Parameters and Validation

Each source image was spatially partitioned into non‐overlapping halves. Training patches were extracted exclusively from one half, and validation and test patches from the other, with an 80/10/10 train/validation/test ratio applied within these spatial partitions using a fixed random seed. This separation ensures that no training patch shares pixel content with any evaluation patch, preventing information leakage between splits. All models were trained with AdamW optimizer (learning rate 1 × 10^−4^, weight decay 1 × 10^−5^), cosine annealing scheduler, combined L1 and SSIM loss (0.9 L1 + 0.1 SSIM), and early stopping with patience of 15 epochs for up to 100 total epochs (Table S3). We report full‐image PSNR (dB) and SSIM as primary metrics, computed by reconstructing full 256 × 256 images from overlapping patch predictions with stride 16 and overlap averaging.

### 4.12 Computational Environment

All models were implemented in PyTorch 2.9. Training and inference were performed on a single Intel Arc 140V GPU 16 Gb. Inference time per image patch was measured as the average of 100 forward passes at batch size 1 after an initial warmup of 10 iterations.

## Author Contributions

**Yunseok Kim** conceived the original idea and supervised this work. **Jaeuk Sung**, **Hongseok Kim**, **Seungjae Heo**, **Jin Tae Moon**, **Jangwoo Ko**, and **Panithan Sriboriboon** performed the experimental measurements. **Jaeuk Sung** and **Jin Tae Moon** contributed to the Python code. **Dong‐Gyu Lee**, **Sanghyun Jo**, **Hee Han**, **Jungho Ryu**, **Woo Lee**, **Jinseong Heo**, and **Hyun‐Cheol Song** prepared the sample. All authors discussed the results and contributed to the paper.

## Conflicts of Interest

The authors declare no conflicts of interest.

## Supporting information




**Supporting File**: advs78289‐sup‐0001‐SuppMat.docx.

## Data Availability

The data that support the findings of this study are available on request from the corresponding author. The data are not publicly available due to privacy or ethical restrictions.
