# Supplementary material for: Physics‐Guided Multi‐Channel Restoration for Efficient and Accurate Atomic Force Microscopy Imaging
Source: Adv Sci (Weinh). 2026 Oct 18:e78289. Online ahead of print. doi: 10.1002/advs.78289 (PMC13677073; doi:10.1002/advs.78289)
Supplement: Supplementary file 1 [file ADVS-9999-e78289-s001.docx]

**Supporting Information**

**Physics-guided multi-channel restoration for efficient and accurate atomic force microscopy imaging**

*Jaeuk Sung^#^, Hongseok Kim^#^, Seungjae Heo^#^, Jin Tae Moon, Jangwoo Ko, Panithan Sriboriboon, Dong-Gyu Lee, Sanghyun Jo, Hee Han, Jungho Ryu, Woo Lee, Jinseong Heo, Hyun-Cheol Song, Yunseok Kim^*^*

Dr. J. Sung, H. Kim, S, Heo, J. T. Moon, J. Ko, Dr. P. Sriboriboon, Prof. Y. Kim

School of Advanced Materials Science and Engineering, Sungkyunkwan University, Suwon 16419, Republic of Korea

Dr. J. Sung

Department of Applied Data Science, Sungkyunkwan University (SKKU), Suwon 16419, Republic of Korea

J. T. Moon, J. Ko

Department of Semiconductor and Display Engineering, Sungkyunkwan University (SKKU), Suwon 16419, Republic of Korea

D. Lee, Prof. H.-C. Song

Department of Materials Science and Engineering, Korea University, Seoul 02841 Republic of Korea

Dr. S. Jo, Dr. J. Heo

Samsung Advanced Institute of Technology, Suwon, 16678 Republic of Korea

Dr. H. Han

National Nano Fab Center (NNFC), Daejeon, 34141 Republic of Korea

Prof. J. Ryu

School of Materials Science and Engineering, Yeungnam University, Gyeongsan, Gyeongbuk 38541, Republic of Korea

Dr. W. Lee

Korea Research Institute of Standards and Science (KRISS), Daejeon, 34113 Republic of Korea

\# These authors contributed equally to this work.

* E-mail: yunseokkim@skku.edu (Prof. Y. Kim)

**1. DnCNN and NAFNet-based U-Net architecture**

Figure S1. Multi-channel input design for AFM image restoration. (a) Schematic of the DnCNN architecture with residual learning. The model takes multi-channel input and outputs restored topography. (b) Four input channels derived from AFM measurement physics: trace topography (primary surface measurement), trace–retrace topography difference (scan direction dependent parachuting and collision artifacts), trace error signal, and retrace error signal (feedback residual providing high-frequency features missed by the feedback loop). All scale bars are 2 μm.

Figure S2. (a) NAFNet-based U-Net architecture used for EFM and PFM cross-modal experiments. The encoder-decoder architecture with attention mechanisms provides multi-scale feature extraction, making it suitable for spatially distributed degradation patterns characteristic of EFM and PFM signals. (b) Representative EFM and PFM input and ground truth images. For EFM image restoration, amplitude and error images are used as inputs to restore amplitude images acquired at scan speed of 3.0 Hz. For PFM image restoration, phase and amplitude images are used as inputs to restore phase image acquired at scan speed of 9.77 Hz and time constant of 0.1 ms.

For AFM topography experiments, we adopted a DnCNN architecture with residual learning (Figure S1(a)). The network consists of stacked convolutional layers, each followed by batch normalization and ReLU activation. The first convolutional layer accepts the multi-channel input (single, two, or four channels) and extracts initial feature maps, while intermediate layers progressively refine these features through repeated convolution-batch normalization-ReLU blocks. The final layer produces a single channel residual map through a sigmoid activation, which is added to the input trace topography via a skip connection to generate the restored image. This residual learning strategy allows the network to focus on learning the artifact component rather than the full surface topography, improving training stability and convergence. Figure S1(b) illustrates the four input channels derived from AFM measurement physics. The trace topography provides the primary surface measurement acquired during the forward scan. The trace-retrace topography difference highlights scan-direction-dependent artifacts, exhibiting large values at step edges where parachuting and collision artifacts differ between forward and backward scans and approaching zero on flat regions where both scan directions record equivalent topography. The trace and retrace error signals capture feedback residuals that encode high-frequency surface features not recorded in the topography channel. Four model sizes were evaluated to assess the interaction between channel configuration and model capacity: tiny (8 layers, 32 features, 57K parameters), small (12 layers, 48 features, 210K parameters), mid (17 layers, 64 features, 558K parameters), and large (20 layers, 96 features, 1,501K parameters). Adding three input channels modifies only the first convolutional kernel, increasing total parameters by less than 1.5% even for the smallest model. This ensures that performance differences between single channel and four channel configurations arise from the additional physical information provided by auxiliary channels rather than from increased model capacity.

For EFM and PFM experiments, we adopted a nonlinear activation free network (NAFNet)-based U-Net architecture rather than the DnCNN used for topographic denoising. (Figure S2) This choice reflects the distinct degradation characteristics of these modalities. Topographic artifacts from feedback loop limitations are spatially localized at step edges, making them well-suited to the feedforward DnCNN architecture. In contrast, EFM and PFM signals suffer from non-topographic degradation sources, including electronic noise from the lock-in amplifier, time constant effects, and scan-speed-induced distortions, that produce spatially distributed patterns across the entire image. The encoder-decoder structure of the NAFNet-based U-Net, with its multi-scale feature extraction and attention mechanisms, is better suited to capture and correct these distributed degradation patterns. The architecture accepts multi-channel inputs (single, two, or four channels) and outputs the restored signal through a skip connection from the input trace channel. Five model sizes from 3.62M to 18.27M parameters were evaluated to assess the interaction between channel configuration and model capacity. For EFM, amplitude trace-retrace and error signal trace-retrace images were used for multi-channel input. For PFM, phase trace-retrace and amplitude trace-retrace images were used for multi-channel input.

**2. AFM feedback loop artifacts at high scan speeds**

Figure S3. Artifacts from high scan speed AFM topographic image acquired at scan speed 7.0 Hz. (a) Trace topography and corresponding trace error signal acquired at high scan speed. The error signal captures feedback residual information not captured by topography alone. (b) Line profiles of the ground truth, input trace topography, and trace error signal. The spatial distribution of parachuting and collision artifacts is clearly visible in the error signal, demonstrating that auxiliary channels encode artifact location and magnitude information critical for restoration. All scale bars are 2 μm.

The AFM feedback controller adjusts the cantilever height to maintain constant tip–sample interaction during scanning. At high scan speeds, the finite bandwidth of the feedback loop prevents the controller from tracking rapid topographic changes, producing two characteristic artifacts. Collision artifacts occur at ascending step edges where the tip impacts the surface before the feedback raises the cantilever, generating overshoot in the topography signal and a large positive error spike. Parachuting artifacts occur at descending step edges where the tip loses surface contact and travels above the true surface, generating an undershoot in topography and a negative error deflection. Because the tip encounters edges from opposite directions during forward (trace) and backward (retrace) scans, these artifacts are directionally complementary: a collision on the trace corresponds to a parachuting event on the retrace at the same edge location. This complementarity provides the physical basis for the multi-channel input design described in the main text.

**3. Representative images of PFM and EFM images measured at different conditions**

Figure S4. Representative PFM and EFM ground truth and input images acquired under different conditions for image restoration. (a) PFM phase and amplitude images with time constants of 0.1 ms and 3.82 ms. (b) PFM phase and amplitude images with scan speeds of 0.1 Hz and 9.77 Hz. (c) EFM amplitude images with scan speed of 0.1 Hz and 3.0 Hz. All scale bars are 2 µm.

Representative images of PFM and EFM ground truth and input images were taken at different conditions (Figure S4). The PFM input images for varying time constant were taken at 0.1 ms time constant compared to ground truth 3.823 ms. The PFM input images for varying scan speed were taken at 9.77 Hz compared to ground truth 0.1 Hz. The EFM input images for varying scan speed were taken at 3.0 Hz compared to ground truth 0.1 Hz. The acquired data were used to demonstrate that the feasibility of multi-channel input image restoration strategy can be applied to different AFM modalities beyond simple topographic image restoration.

**4. Training convergence for multi-channel configurations**

Figure S5. Training and validation loss curves for single channel, two channel, and four channel DnCNN at mid size (558K parameters). Multi-channel models converge to lower final loss values and exhibit faster initial convergence compared with the single channel baseline.

Figure S5 shows the training and validation loss curves for the three channel configurations using the mid-size DnCNN architecture. The four channel model converges to the lowest final loss and reaches its plateau approximately 20 epochs earlier than the single channel model. The two channel model shows intermediate behavior, consistent with the monotonic improvement in PSNR and SSIM reported in the main text. The faster convergence of multi-channel models is expected. By providing explicit artifact information through additional channels, the network requires fewer training iterations to learn the mapping from degraded to clean images. The validation curves closely track the training curves throughout the training process, indicating no overfitting across all channel configurations.

**5. Image restoration examples across test images**

Figure S6. Additional visual comparisons of single channel and four channel DnCNN reconstructions across all test images restored from input image acquired at scan speed 7.0 Hz. The four channel model consistently recovers sharp step edges and fine surface features across diverse sample morphologies, whereas the single channel model produces blurred edges.

To verify the consistency of the multi-channel advantage, Figure S6 presents restoration results for all test images not shown in the main text. Across all samples, the four channel DnCNN produces reconstructions closely matching the ground truth, particularly at step edges and fine topographic features. The single channel model, while recovering the overall surface morphology, consistently produces blurred edges and fails to resolve features below the feedback bandwidth limit. These results confirm that the performance improvement reported in Figure 4 is representative of the entire test set and is not limited to specific sample morphologies.

**6. Multi-channel gain as a function of artifact severity**

Figure S7. PSNR and SSIM gain of four channel model over single channel DnCNN as a function of input scan speed (1.0 to 7.0 Hz, mid size DnCNN). The advantage of the multi-channel input increases with artifact severity, reaching +5.71 dB at the highest scan speed.

Figure S7 quantifies the multi-channel performance advantage as a function of scan speed. At 1.0 Hz, where artifacts are minimal and the feedback loop tracks the surface adequately, the four channel gain is small (+0.14 dB) because all channels contain similar low-artifact information. As scan speed increases to 2.0 Hz, 5.0 Hz, and 7.0 Hz, the multi-channel gain grows to +1.37 dB, +3.21 dB, and +5.71 dB, respectively. The SSIM gain follows the same monotonic trend. This scaling behavior is physically intuitive. As the feedback loop fails progressively at higher scan speeds, the error signal amplitudes increase and the trace-retrace discrepancy becomes more pronounced, making the auxiliary channels increasingly informative. This property is particularly relevant for high-throughput semiconductor metrology applications, where scan speeds of 5 Hz and above are desirable for wafer-scale characterization.

**7. Quantification of each channel contribution to topographic image restoration**

Figure S8. Full factorial analysis of auxiliary channel contributions to topographic AFM restoration at 7.0 Hz. (a) PSNR for all eight channel subsets retaining trace topography (T). trace–retrace, topography difference (D), trace error (Et), and retrace error (Er). (b) Exact Shapley contributions of the three auxiliary channels, calculated from the PSNR values.

All eight combinations of the trace–retrace topography difference (D), trace error (E_t_), and retrace error (E_r_) were evaluated while retaining trace topography (T) as the primary input using DnCNN architecture. The PSNR increased from 21.28 dB for T alone to 25.57 dB for the complete input. Removing D, E_t_, or E_r_ reduced PSNR to 24.80, 24.75, and 24.53 dB, respectively. Exact Shapley contributions, calculated from the eight configurations, were 1.35 dB for D, 1.24 dB for E_t_, and 1.70 dB for E_r_.

**8. Input-dimensionality control experiment**

Figure S9. Input-dimensionality controls for topographic AFM restoration at 7.0 Hz. The image restoration PSNR is compared for the single channel DnCNN baseline, four channel input, four repeated copies of the primary topography, mismatched auxiliary signals, and Gaussian noise replacing the auxiliary signals with matched means and standard deviations. All four channel configurations use the same DnCNN architecture.

To examine whether increased input dimensionality alone accounted for the restoration gain, three control configurations were evaluated using the four channel DnCNN architecture. These comprised four repeated copies of trace topography, mismatched auxiliary signals, and Gaussian noise matched to the auxiliary channel means and standard deviations. These controls achieved PSNR values of 21.36, 19.90, and 20.12 dB, respectively, compared with 25.57 dB for the spatially corresponding physical four channel input and 21.28 dB for the single channel baseline (Figure S9). The repeated-primary control performed similarly to the single channel baseline, while mismatched and noise auxiliary inputs reduced performance. These results support the importance of spatially corresponding measurement information in obtaining the observed restoration gain.

**9. Structural fidelity and hallucination evaluation of restored images**

Figure S10. Step-boundary preservation in a topographic AFM images acquired from a micro-patterned structures at 7.0 Hz, using a 0.5 Hz measurement as the reference. The upper row shows the raw input, single channel reconstruction, four channel reconstruction, and reference. The middle row shows extracted boundaries, and the lower row shows direct boundary overlays. Boundary F1 was evaluated with a two-pixel tolerance. All scale bars are 2 µm.

Figure S11. Domain-wall preservation in PFM phase images of KBT-BT-NBT acquired at 9.77 Hz, using a 0.1 Hz measurement as the ground truth. Models trained for the 9.77 Hz condition were used for reconstruction. The upper, middle, and lower rows show phase images, extracted domain walls, and direct reference-boundary overlays, respectively. Boundary distances are reported in pixels, and boundary F1 uses a two-pixel matching tolerance. All scale bars are 1 µm.

Figure S12. Feature-boundary preservation in EFM amplitude images of nanocapacitor acquired at 3.0 Hz, using a 0.1 Hz measurement as the reference. The upper row shows amplitude images, the middle row shows extracted feature boundaries, and the lower row shows direct reference-boundary overlays. Segmentation was based on the absolute deviation from the median amplitude using a reference-derived threshold. Boundary distances are reported in pixels, and boundary F1 uses a two-pixel matching tolerance. All scale bars are 2 µm.

Figure S13. Quantitative boundary comparison for the selected patches shown in Figures S10–S12. (a) Symmetric mean boundary distance in pixels. (b) Boundary F1 score evaluated with a two-pixel matching tolerance. Raw inputs, single channel reconstructions, and four channel reconstructions are compared for each modality.

Structural fidelity was assessed for topographic AFM, PFM phase, and EFM amplitude images (Figures S10–S12). Segmentation thresholds were determined from the corresponding low-speed reference patches using Otsu’s method and applied consistently to the raw and restored images. Symmetric boundary distance was calculated as the average of the mean predicted-to-reference and reference-to-predicted boundary distances. Boundary precision, recall, and F1 were evaluated with a two-pixel matching tolerance. The image restoration process reduced the symmetric mean boundary distance and increased the boundary F1 score, with the four channel reconstructions showing the closest agreement with the references (Figure S13).

**10. Additional PFM image reconstruction evaluation**

Figure S14. PFM phase-image restoration of a polycrystalline lead zirconate titanate (Poly-PZT) thin film at different lock-in time constants. Rows show input time constants of 0.1, 0.5, 1.003, and 1.973 ms at a scan rate of 0.5008 Hz. Columns show the raw phase-trace input, single channel reconstruction, four channel reconstruction, and the corresponding reference acquired at 3.823 ms. The single channel model uses phase trace, whereas the four channel model uses phase trace/retrace and amplitude trace/retrace. Four channel reconstruction improves correspondence with the reference. Scale bars, 1 μm.

Figure S15. Quantitative comparison of the selected Poly-PZT phase-image reconstructions shown in Figure R6. (a) PSNR, (b) SSIM, and (c) Pearson correlation coefficient relative to the corresponding ground truth reference (3.823-ms time constant). Black, red, and blue bars represent the raw input, single channel reconstruction, and four channel reconstruction, respectively.

Additional PFM phase-image restoration experiments were conducted on a polycrystalline lead zirconate titanate (PZT) thin film. Images were acquired over 5 × 5 μm² fields with 256 × 256 pixels at a scan rate of 0.5008 Hz. Input time constants were 0.1, 0.5, 1.003, and 1.973 ms, and the corresponding 3.823-ms acquisition was used as an operational reference. Single channel models used phase trace, whereas four channel models used amplitude trace/retrace and phase trace/retrace. Figure S14 presents direct full-image reconstructions for fields associated with the condition-wise highest-SSIM four channel patches. Figure S15 reports phase-image PSNR, SSIM, and Pearson correlation for these selected full images against their corresponding ground truth image. Four channel reconstruction improved all three metrics relative to single channel reconstruction. Nevertheless, incomplete correspondence of fine domain features remained, including at the longer input time constants.

**11. Data efficiency across AFM modalities**

Figure S16. PSNR as a function of training data fraction for single channel and four channel models across all three AFM modalities: EFM amplitude, PFM scan speed, and PFM time constant. The four channel model consistently outperforms the single channel model at all data fractions, with the advantage amplified at low data fractions.

We systematically evaluated data efficiency by training the smallest model size (3.62M parameters) at 12.5%, 25%, 50%, 75%, and 100% of available training patches for each channel configuration and modality (Figure S16). The four channel model requires substantially less training data to achieve equivalent performance to the single channel model across all three experimental conditions. For EFM amplitude restoration, four channel at 25% data matches single channel at approximately 53% data (2.1× efficiency). For PFM scan speed degradation, four channel at 12.5% data matches single channel at approximately 37% data (3.0× efficiency). For PFM time constant degradation, four channel at 25% data matches single channel at approximately 64% data (2.6× efficiency). This data efficiency advantage is practically significant. Collecting slow-scan reference images for training is time-consuming, and reducing the required dataset size by a factor of two to three substantially lowers the barrier to deploying restoration on new sample types or measurement configurations.

**12. Computation efficiency across AFM modalities**

Figure S17. PSNR as a function of model parameters for single channel and four channel models across all three AFM modalities. Smaller four channel models consistently match or exceed the performance of larger single channel models, demonstrating approximately 1.8 to 2× parameter reduction.

Computation efficiency was evaluated by training models at five sizes (3.62M to 18.27M parameters) for each channel configuration across all three modalities (Figure S17). In every case, a smaller four channel model matches or exceeds a larger single channel model at the same quality level. For EFM, four channel at 9.4M parameters outperforms single channel at 18.3M parameters. For PFM under both scan speed and time constant degradation, a four channel model with 3.62M parameters achieves quality comparable to a single channel model with 6.37M parameters, representing approximately 1.8× parameter reduction. These results confirm that multi-channel input externalizes information the single channel model must otherwise encode in additional parameters. When trace and retrace signals are both available as input, the network does not need to learn scan-direction-dependent artifact patterns from topography alone. This reduces the required model capacity for a given restoration quality.

**13. Effectiveness of multi-channel input at different architecture**

Figure S18. PSNR and SSIM comparison between single- and four channel configurations of Physics-gated DnCNN, U-Net, and parameter-matched Restormer for topographic AFM restoration at 7.0 Hz.

Figure S19. Example figure of 7.0 Hz topographic AFM image restored with 1 channel and 4 channel DnCNN, U-Net, and Restormer. All scale bars are 2 µm.

Single- and four channel configurations were compared across the evaluated restoration architectures using PSNR and SSIM (Figure S18). PSNR increased from 22.10 to 27.48 dB for DnCNN, from 23.01 to 25.65 dB for U-Net, and from 22.91 to 26.86 dB for the parameter-matched Restormer. SSIM increased from 0.82 to 0.90 for DnCNN, from 0.83 to 0.86 for U-Net, and from 0.84 to 0.88 for the parameter-matched Restormer. For all three architectures, the multi-channel input showed stronger image restoration performance compared to single channel input.

**14. EFM and PFM image restoration under limited data counts**

Figure S20. EFM amplitude trace restoration at varying data fractions (12.5–100%) for restoration of image acquired at scan speed 3.0 Hz. The four channel model maintains high reconstruction quality even at severely reduced data fractions, whereas the single channel model exhibits poor image restoration at low data fractions. All scale bars are 1 μm.

Figure S21. PFM scan speed phase restoration at varying training data fractions for restoration of image acquired at scan speed 9.77 Hz. four channel model preserves domain structures across all data conditions, whereas single channel model shows noisy reconstructions at low data fractions. All scale bars are 1 μm.

Figure S22. PFM time constant phase restoration at varying data fractions for image acquired at 0.1 ms time constant. four channel model preserves domain structures across all data conditions, whereas single channel model exhibits noisy reconstructions at low data fractions. All scale bars are 1 μm.

Domain structure image reconstruction for EFM and PFM demonstrates higher data efficiency for the four channel model. Figure S20 presents EFM amplitude image restoration results under scan speed degradation, where input images were acquired at 30 times the ground truth scan speed. The high scan speed introduces motion-induced artifacts and spatial distortion that obscure the underlying electrostatic domain structure. The four channel model successfully recovers domain boundaries at 75% data availability, whereas the single channel model fails to produce adequate restoration even at 100% data availability. The PFM phase domain structure image reconstruction for low time constant and high scan speed shows similar results. For scan speed degradation (Figure S21), the four channel model preserves domain structures that are progressively lost by the single channel model as the training data fraction decreases. Even at 25% training data, the four channel model produces interpretable domain images, while the single channel model at the same fraction produces visibly degraded reconstructions. For time constant degradation (Figure S22), where the input images were acquired at 0.1 ms time constant versus 3.823 ms ground truth, the four channel model again demonstrates superior preservation of domain features. The consistent advantage across both degradation types confirms the multi-channel input provides complementary information regardless of the specific noise source.

**15. PFM image restoration for different model sizes**

Figure S23. PFM time constant computation efficiency test result comparing between four channel model with 3.62M parameters and single channel model with 6.37M parameters for restoration of image acquired at 0.1 ms time constant. The smaller four channel model achieves comparable reconstruction quality while using fewer parameters. All scale bars are 1 μm.

Figure S23 provides detailed image reconstruction results from the PFM computation efficiency analyses. The time constant degradation shows consistent four channel superiority with approximately 1.8× parameter reduction at matched quality. These results demonstrate that the parameter efficiency advantage of multi-channel input is robust across modalities and is not specific to the topographic denoising task.

**Table S1.** Scanning and image restoration conditions for topography, PFM, and EFM experiments.

| **Parameter** | **Topography denoising**  **(scan speed)** | **PFM denoising**  **(time constant)** | **PFM denoising**  **(scan speed)** | **EFM denoising**  **(scan speed)** |
| --- | --- | --- | --- | --- |
| Gain | 1.0 | 1.0 | 1.0 | 1.0 |
| Pixles | 256 × 256 | 256 × 256 | 256 × 256 | 256 × 256 |
| Scan size | 10 × 10 µm 15 × 15 µm  30 × 30 µm | 10 × 10 µm | 10 × 10 µm | 10 × 10 µm |
| GT condition | Scan speed 0.5 Hz | Time constant 3.823 ms | Scan speed 0.1 Hz | Scan speed 0.1 Hz |
| Input condition | Scan speed 7.0 Hz | Time constant 0.1 ms | Scan speed 9.77 Hz | Scan speed 3.0 Hz |
| Available signals | Error signal trace / retrace  Topography trace / retrace | Phase trace / retrace  Amplitude trace / retrace | Phase trace / retrace  Amplitude trace / retrace | Error signal trace / retrace  Amplitude trace / retrace |
| Deep learning architecture | DnCNN | NAFNet | NAFNet | NAFNet |
| Model size | 57k, 210k, 558k, 1,501k | 3.62M, 6.37M, 12.74M, 18.27M | 3.62M, 6.37M, 12.74M, 18.27M | 3.62M, 6.37M, 12.74M, 18.27M |

Table S1 summarizes the scanning conditions and image restoration configurations for all experiments. Topographic AFM measurements were performed using an NX-10 microscope with a Multi75E-G probe in non-contact mode. PFM measurements were performed using an MFP3D microscope with a PPP-NCHPt probe. EFM measurements were performed using an FX40 microscope with a PPP-EFM probe in dual-pass lift mode at a lift height of 50 nm. The input/reference scan rates were 7.0/0.5 Hz for topographic AFM, 9.77/0.1 Hz for the PFM scan- rate experiment, and 3.0/0.1 Hz for EFM. For the PFM time-constant experiment, the input and reference time constants were 0.1 and 3.823 ms, respectively. Feedback gain values are reported as instrument settings. For topographic AFM image reconstruction, images were acquired at scan sizes ranging from 10 × 10 to 30 × 30 µm with 256 × 256 pixels. Ground truth images were collected at 0.5 Hz scan speed, and input images were acquired at 7.0 Hz to introduce feedback artifacts. Available signals include error signal trace/retrace and topography trace/retrace, which were processed using the DnCNN architecture at four model sizes (57K to 1,501K parameters). For PFM denoising, two degradation conditions were examined: time constant variation (ground truth 3.823 ms, input 0.1 ms) and scan speed variation (ground truth 0.1 Hz, input 9.77 Hz). Both PFM conditions used phase trace/retrace and amplitude trace/retrace as available signals. For EFM denoising, amplitude images were acquired at 0.1 Hz (ground truth) and 3.0 Hz (input), with error signal trace/retrace and amplitude trace/retrace as available signals. All PFM and EFM experiments used the NAFNet-based U-Net architecture at five model sizes (3.62M to 18.27M parameters). A feedback gain of 1.0 was maintained across all experiments to ensure consistent imaging conditions.

**Table S2.** Data augmentation methods for all experimental conditions.

| **Parameter** | **Topography denoising**  **(scan speed)** | **PFM denoising**  **(time constant)** | **PFM denoising**  **(scan speed)** | **EFM denoising**  **(scan speed)** |
| --- | --- | --- | --- | --- |
| Original image pairs | 11 | 5 | 5 | 5 |
| Patch division | 256 × 256 → 64 × 64 | 256 × 256 → 64 × 64 | 256 × 256 → 64 × 64 | 256 × 256 → 64 × 64 |
| Stride | 32 | 32 | 32 | 32 |
| Total patch images for training | 539 | 245 | 245 | 245 |
| Train : Validation : Test | 431 : 54 : 54 | 196 : 24 : 25 | 196 : 24 : 25 | 196 : 24 : 25 |
| Augmentation | Horizontal flip, Vertical flip,  90º rotation (0, 90, 180, 270) | Horizontal flip, Vertical flip,  90º rotation (0, 90, 180, 270) | Horizontal flip, Vertical flip,  90º rotation (0, 90, 180, 270) | Horizontal flip, Vertical flip,  90º rotation (0, 90, 180, 270) |
| Images after augmentation | 6896 : 54 : 54 | 3136 : 24 : 25 | 3136 : 24 : 25 | 3136 : 24 : 25 |

Dataset composition for all experiments. For topographic denoising, 11 source image pairs were augmented to 6,896 patches. For EFM and PFM, 5 source image pairs per condition were augmented to 3,136 patches. All patches are 64 × 64 pixels extracted with stride 32. To prevent information leakage between training and evaluation sets, each 256 × 256 source image was spatially partitioned into non-overlapping halves. Training patches were extracted exclusively from one half, and validation and test patches from the other half. This spatial separation ensures no training patch shares pixel content with any evaluation patch, providing a conservative estimate of generalization performance. A fixed random seed was used for all partitioning and augmentation to ensure reproducibility.

**Table S3.** Deep learning training parameters

| **Parameter** | **Value** |
| --- | --- |
| Optimizer | AdamW  (learning rate=10^-4^, weight decay=10^-5^) |
| Scheduler | Cosine annealing |
| Max epochs | 100 |
| Early stopping | Patience=15 |
| Patch size | 64×64 |
| Stride (train) | 32 |
| Loss | 0.9 × L1 + 0.1 × SSIM |

All models were trained with AdamW optimizer (learning rate 1 × 10⁻⁴, weight decay 1 × 10⁻⁵), cosine annealing scheduler, combined L1 and SSIM loss (0.9 L1 + 0.1 SSIM), early stopping with patience of 15 epochs, for up to 100 total epochs.
